# The Role of *CD14* and *CTLA4* Gene Polymorphisms in Risk of Celiac Disease among Patients of Iranian Ethnicity

**Published:** 2014-05-25

**Authors:** Mahdi Zamani, Fatemeh Karami, Fariba Shirvani, Laleh Kia-Lashaki, Bizhan Shahbazkhani

**Affiliations:** 1 Department of Medical Genetics, Faculty of Medicine, Tehran University of Medical Sciences, Tehran, Iran; 2 Department of Neurogenetics, Iranian Center of Neurological Research , Tehran University of Medical Sciences, Tehran, Iran; 3 Department of Pediatrics, Pediatric Infections Research Center, Mofid Children Hospital. Shaheed Beheshti University of Medical Sciences and Health Services, Tehran, Iran; 4 Digestive Disease Research Institute, Shariati Hospital, Tehran University of Medical Sciences, Tehran, Iran

**Keywords:** *CTLA4*, *CD14*, Celiac disease

## Abstract

**Objective:**

Celiac disease (CD) is developed via autoimmune reactions against gluten which is mainly found in grains. Although HLA DQB1 locus is the most important genetic susceptibility to CD, some other variants such as *A49G* and *G1359T* of *CTLA4* and *CD14* genes respectively have been proposed as CD predisposing genetic factors in many vari- ous studies. We aimed to assess possible roles of *A49G* and *G1359T* polymorphisms in CD susceptibility in the Iranian population.

**Materials and Methods:**

In this case-control, one hundred CD patients and 100 healthy matched controls with average age of 30-33 years were selected. They were genotyped for both *A49G* and *G1359T* polymorphisms using polymerase chain reaction-restriction fragment length polymorphism (PCR-RFLP).

**Results:**

There was no association between genotypes of *A49G* variant of *CTLA4* and risk of CD (p<0.05). The *G1359T* polymorphism of *CD14* gene also did not show any significant association with risk of CD among the studied population. However, patients with *CD14* T/T genotype were more classified in the severe form (Marsh III) of CD, showing border line significance (p<0.05).

**Conclusion:**

No association was identified between the combination of 1359T and *A49G* alleles with risk of CD. These lacks of association could be due to small sample size and considering further studies in various populations and ethnicities seems to be required.

## Introduction

In genetically predisposed individuals, ingestion of food containing gluten protein drives some immunological and allergic responses which can lead to malabsorption, diarrhea, weight loss and some other morbidity in a disorder called celiac disease (CD). Consumption of gluten rich food including all types of grains, results in gastrointestinal irritation and consequent colon atrophy (1). The prevalence of CD is around 1 and 0.6 % among Western Europe and Iranian populations, respectively (2, 3). CD patients are also susceptible to infertility due to oligo-ovulation, malabsorption and hyperor hypothyroidism (4). Various autoimmune disorders such as coagulopathies due to insufficiency of vitamin k, recurrent abortion, primary biliary cirrhosis, microscopic colitis and diabetes mellitus type 1 (T1DM) are also more observed in CD patients (5, 6). Thus, differential diagnosis of CD patients has become more elusive and needs robust biochemical and molecular tests including tests of genetic markers.

Currently, diagnosis of CD is usually based on serological tests including detection of antibodies against tissue transglutaminase (tTGA) and Endomysium, and colon biopsy which all are expensive and accompany false positive and negative results (7). Investigation on human leukocyte antigen (HLA) class II locus led to the identification of a strong association between CD and HLA DQ locus including DQ-A1 05, DQ-B1 0201, DQB1 0302 and DQ-A1 03 (8). Moreover, there are some other reports concerning the roles of CD28/ *CTLA4*/ ICOS genetic locus found on 2q33 along with 5q31-33 encoding *CD14* in development of CD (9).

Cytotoxic T-lymphocyte associated antigen 4 (*CTLA4*) controls T cell activation and proliferation through releasing negative co-stimulatory signals and plays critical roles in making tolerance to self antigens (10). It was demonstrated that some variants in *CTLA4* gene are associated with autoimmune diseases like Graves ‘disease and T1DM (11). *A49G* is one of these variants residing in exon 1 and has shown meaningful association with CD enteropathy in specific populations which will be mentioned in the discussion section.

*CD14* cytokine is an active member of innate immunity and is involved in the clearance of apoptotic cells. It was described that the *G1359T* polymorphism of *CD14* gene is associated with over-expression of this cytokine (12). On the other hand, it was seen that *CD14* was also upregulated in individuals suffering from inflammatory bowel disease (IBD) (13). However, the contribution of *G1359T* polymorphism in the pathogenesis of CD remains to be explained. Therefore, the present work was conducted to estimate the frequency of *G1359T* and *A49G* variants in a subset of Iranian population and find out their contribution to CD pathogenesis in unrelated affected individuals.

## Materials and Methods

One hundred CD patients (41 males and 59 females) were selected from the databank of Iranian CD patients Association and were completely matched with 100 healthy controls especially for ethnicity (Farsi speaking), sex and age, to participate in the present case-control association study. Questionnaires including age, gender, familial history, medical and drug history, etc. were filled for all the enrolled cases and controls. Mean of age was 33 and 30 years in cases and controls, respectively. The diagnosis of CD was based on biopsy and positive reaction to gluten free diet. For all cases and control samples, 5 mL of blood were taken and then transferred into canonical tubes containing 200 μL EDTA.

### Ethical considerations

All the enrolled patients and controls filled the consent form according to the protocol of the Ethical Review Board of Tehran University of Medical Sciences.

### DNA extraction

DNA extraction was carried out from all blood samples using modified salting out method (14) and the quality and concentration of isolated genomic DNA were determined through loading on agarose (1%) gel electrophoresis and spectrophotometry.

### Polymerase chain reaction-restriction fragment length polymorphism (PCR-RFLP) for genotyping *G1359T* polymorphism of *CD14*

The *G1359T* polymorphism residing in exon 1 of *CD14* was genotyped through amplification in PCR reaction using appropriate primers (forward 5´ AGCACTTTGAGAGGCCAAGG C 3´ and reverse 5´ CCACATCCCTAGACCTCTGGG 3´). The primers were those reported by Bonitto et al. (15) and were verified again using Primer- BLAST in NCBI web site. 100 ng of genomic template was added to the PCR mix (25 μl) including 2.5 μl of, 12.5 pmol of each forward and reverse primers, 2.5 μl of 10x buffer including 1.5 mM Mgcl_2_, 0.2 mM of dNTP mixture and 1U of Taq DNA polymerase (Cynagen, Iran). After an initial denaturation at 94˚C for 5 minutes, 30 cycles of PCR was performed according to the following program in a thermocycler (Bio-Rad Laboratories, Hercules, CA, USA), 30 seconds at 94˚C for denaturation, 30 seconds at 56˚C for annealing, 30 second at 72˚C for extension and a final extension at 72˚C for 5 minutes. The PCR products were then digested through incubation at 55˚C with BseGI restriction endonuclease (Sigma, USA) for one hour to determine the presence of the T allele of *CD14* gene. The 6-10 μL of every PCR product was mixed with 0.5 μL of BseGI enzyme and 1.2 μL of its specific buffer and then the reaction mixture was adjusted to 12 μL by ddH_2_O. Digested fragments were detectable after loading on a polyacrylamide gel (6%) and subsequent staining of gel using ethidium bromide. The products of digestion were 208 bps and 95 bps fragments in the presence of TT genotype and only one band corresponding to the PCR product (303 bps) when the genotype was GG.

### PCR-RFLP for genotyping A49G polymorphism of CTLA4 gene

The required primer sequences for amplification designed by Primer3 program were used in the following PCR reaction: 12.5 pmol of each forward (5´ GCTCTACTTCCTGAAGACCT 3´) and reverse primer (5´ AGTCTCACTCACCTTTGCAG 3´), 2.5 μl of 10x buffer including 1.5 mM Mgcl_2_, 0.2 mM of dNTP mixture and 1 U of Taq DNA polymerase (Cynagen, Iran) and 100 ng of genomic DNA. The PCR program was similar to *CD14* gene amplification except for the annealing temperature which was optimized at 58˚C. Afterward, 0.5 μl of Sat I restriction enzyme (Sigma, USA) along with 1.2 μl of Green buffer and ddH_2_O were used to digest 6-10 μl of each PCR products. The digestion products were separated on polyacrylamide gel (6%). In the presence of allele G, SatI recognizes two cutting sites and cleaves the 162 bps PCR product into 74, 63, and 25 bps fragments. In the wild type status, SatI produces two 63 and 99 bps fragments and provides an internal control for reaction. Therefore, AG or heterozygote genotype gives 74, 63, 25, and 99 bps fragments.

### Statistical analysis

SPSS version 17 and MS Excel were employed to analyze obtained data. The Hardy–Weinberg equilibrium was assayed in our case and control groups using Pearson’s test. In addition, we utilized the Fisher’s exact test to find the genotypephenotype correlation in our studied population. P-values less than 0.05 have been considered as meaningful and confidence of interval (CI) was taken at 95%.

## Results

### G1359T polymorphism and risk of CD

The frequency of all possible genotypes and alleles regarding *G1359T* polymorphism were determined in case and control groups (Table 1). The frequency of GG, GT and TT genotypes were 63, 32, and 5% in cases and 60, 33, and 7% in controls, respectively. Analysis by means of Pearson’s х^2^ and Fisher’s exact test have shown that there was no significant association between CD and the genotypes and alleles of *G1359T* variant (p>0.05). Moreover, there was no meaningful association between the gender of CD patients and specific 1359 G/T genotypes while GG and TT genotypes were the most and least frequent genotype in both case and control groups.

All of the enrolled patients were classified into five Marsh based on clinical presentation. There were 9, 5, 10, 21, and 15 individuals in Marsh I, Marsh II, Marsh IIIa, Marsh IIIb and Marsh IIIc respectively (data not shown). The prevalence of 1359 G/T polymorphism were compared among different Marshes of CD patients. It was seen that only TT genotypes was in borderline association with Marsh III (p=0.03).

### 49 A/G polymorphism and risk of CD

The frequencies of 58, 36, and 6 % were observed for AA, AG and GG genotypes in cases compared with 61, 35, and 4% in the same genotypes of control group, respectively (Table 1). The observed genotype frequencies of 49A/G variant in our case and control groups had no strong deviation from HW expectations. Indeed, there was no significant association between genotypes and alleles corresponding to 49 A/G polymorphism and risk of CD (p>0.05). In addition, the results of Fisher’s exact test were also not significant in demonstrating higher CD in specific gender carrying either AA, AG or GG genotypes and also G or A alleles.

Calculating the frequency of genotypes and alleles of 49A/G polymorphism in all Marsh of CD (data not shown) demonstrated that *A49G* variant had neither weak nor strong effects on the severity of disease in our patient group.

The combination of 1359 G/T and 49 A/G genotypes did not reveal significant difference between cases and controls (Table 2).

**Table 1:** Frequencies of alleles and genotypes of G1359T polymorphism of CD14 gene and A49G polymorphism of CTLA4 gene in CD patients and controls


Polymorphism	Patients (N=100)	Controls (N=100)	P value

**CD14 -1359G/T **			
**>Genotype frequencies**			
**GG**	60	63	> 0.05
**GT**	33	32	> 0.05
**TT**	7	5	>0.05
**Allele frequencies**			
**G**	153(76.5%)	158(79%)	>0.05
**T**	47(23.5%)	42(21%)	>0.05
**CTLA4-49A/G**			
**Genotype frequencies**			
**AA**	61	58	> 0.05
**AG**	35	36	> 0.05
**GG**	4	6	>0.05
**Allele frequencies**			
**A**	156(76%)	152(78%)	>0.05
**G**	48(24%)	44(22%)	>0.05


P was calculated by Ҳ2 with a 2*2 contingency table.

**Table 2:** Association between CD and combination of both A49G and G1359T polymorphisms in case and control groups


Polymorphism	Patients (N=100)	Controls (N=100)	P value

**Genotype**			
**GG/TT**	2	2	>0.05
**GG/CT**	1	0	>0.05
**GG/CC**	1	4	>0.05
**AA/TT**	3	2	>0.05
**AA/GG**	37	36	>0.05
**AG/TT**	2	1	>0.05
**AA/TG**	11	12	>0.05
**AG/GG**	22	23	>0.05


P was calculated by Ҳ2 with a 2*2 contingency table.

## Discussion

Celiac or gluten-sensitive enteropathy is an autoimmune disorder which is characterized by colon inflammation and atrophy resulting in crypt hyperplasia. This inflammatory cascade is mediated by products of DQB1 and DQA1 loci of HLA class II and subsequent activation of CD4^+^ and CD25^+^ T lymphocytes (8). However, T cell activation is also under the control of costimulatory proteins such as *CTLA4* which is expressed on helper (CD4^+^) and regulatory T cells. *CTLA4* acts as an inhibitory signal and terminates T cell response particularly against self antigens (16). Given the importance of *CTLA4* in autoimmune reactions, the *A49G* (Thereonine to Alanine amino acid exchange) which is the only missense polymorphism recognized in exon 1 of this gene was selected to elucidate the role of it in pathogenesis of CD. In this study, we couldn’t find any meaningful association between all the genotypes and alleles of *A49G* variant and risk of CD. Investigations in most of the other populations could be the further proof on this finding. Our results was in line with the study on 41 Basque families containing CD patients, where there was no significant difference in allele A frequency between case and control groups (17). Concordantly, studying of Tunisian and Italian CD families using maximum likelihood score (MLS) and transmission disequilibrium test (TDT) analyses could not reveal any association and linkage with *A49G* variant and risk of CD (18). The Finnish CD family-based study also found no association and linkage using TDT and affected sib pairs (ASP) analysis within 100 CD families (19). Moreover, the same findings were replicated in a large number of CD families from Britain, Holland, Germany, Sweden and Switzerland (20), which are consistent with our results. van Belzen et al. (21) could not also find any significant difference between cases and controls for the frequency of alleles A and G (p>0.05), although the GG genotype had borderline significance association (p<0.05) among Dutch CD patients (21). In another experiment on 107 Norwegian and Swedish families, the borderline association between 49A/G genotype and severity of CD (p<0.05) was reported. However, there was no more susceptibility to CD in a specific gender carrying *A49G*, which is consistent with our population (22). In another study (23) on 86 cases, 144 control and 113 trios, the frequency of allele A had a significant frequency difference (p=0.03) among Italian CD patients and their closely matched healthy subjects which was in contrast with a previously performed Italian study (18). In addition, it was proposed that allele A is probably associated with CD in those patients who lack HLA DQ2 alleles. Hunt et al. (24) analyzed 340 UK Caucasian patients affected by CD in comparison with 321 normal controls. They demonstrated that allele A of *A49G* variant accompanied by five other polymorphisms in a haplotype was strongly associated with CD (p<0.05). However, it may be due to the effects of the other four polymorphisms within the haplotype. The association of A allele of *A49G* polymorphism was identified in French CD patients (25) which is in contrast with the results of other central European populations (20).

*CD14* encodes a receptor expressed on monocytes and macrophages and attaches to lipopolysacharids of gram negative bacteria and induces activation of mitogen activated protein (MAP) kinase and following pro-inflammatory cytokines (26). *CD14* receptor has low expression in intestine especially in macrophages of lamina propedias to adapt with normal colon flora (27). It was described that the level of *CD14* mRNA is significantly higher in patients suffering from IBD. This may be due to an increase in attachment of *CD14* receptors to gram negative and commensal bacteria and subsequent activation of inflammatory signals. Since inflammation changes the epithelial permeability of intestine, dietary gluten may be accumulated in lamina propedia, where the gluten reactive T cells exist. In addition, *CD14* receptor has a pivotal role in phagocytosis of apoptotic cells and thereby avoids recognition of self antigens and giving rise of autoimmune disorders (28). *G1359T* polymorphism occurs in the promoter region and is associated with higher expression of *CD14*. It was shown that TT genotype of *CD14* gene was linked with high level of serum *CD14* (s*CD14*) compared with CT or CC genotypes (12). Moreover, *G1359T* was associated with lower levels of serum Ig E (13). In the present study, there was no association between genotypes and alleles of *G1359T* polymorphism and risk of CD enteropathy. However, it has been shown that those patients who were classified in Marsh III of CD possessed more TT genotype than other *G1359T* genotypes (data not shown). Boniotto et al. (15) have studied 115 and 37 celiac patients with and without HLA DQ2 or DQ8 predisposing alleles, respectively. Although, there was no association between *G1359T* variant and risk of CD, the TT genotype was more frequent in patients without HLA susceptibility alleles compared with 180 normal volunteers. A recent Italian study on 938 CD patients and 533 controls for investigating the role of three major *CD14* polymorphisms (c.-1145A>G and c.-159C>T, c.-1359G>T) in CD has revealed that only -1145A>G and -159C>T promoter variants had significant association with CD patients who were either carriers of HLA DQ risk alleles or not (29). Our results were consistent with the latter study and demonstrated no association between 1359G/T polymorphism and CD. However, higher frequency of TT genotype was detected in patients classified in Marsh III of CD (data not shown). Matched ethnicity for all of our study population indicates that the absence of association between two *G1359T* and *A49G* variants was not influenced by genetic background and population stratification.

Overall, *A49G* polymorphism has shown different association in various populations which sometimes weighs on no association. It needs doing larger studies including much more samples in different ethnicities of Iranian and other populations.

## Conclusion

To our knowledge, this study primarily showed that *G1359T* and *A49G* polymorphisms as well as their combination were not associated with risk of CD possibly indicating absence of any interaction between them. Given the insufficient published data in Middle East, we can not speculate that the role of these two polymorphisms is ethnic specific. merriting further studies.
